# Onchocerciasis overview: pathogenesis, clinical spectrum, and therapeutic challenges of a neglected tropical disease

**DOI:** 10.3389/fpara.2026.1690973

**Published:** 2026-03-13

**Authors:** Charity N. Njeshi, Alan P. Robertson, Richard J. Martin

**Affiliations:** Department of Biomedical Sciences, Iowa State University, Ames, IA, United States

**Keywords:** drug resistance, mass drug administration, neglected tropical disease, onchocerciasis, pathogenesis, vaccine

## Abstract

Onchocerciasis is still a major health burden in sub-Saharan Africa and Yemen, where many cases occur as control efforts face persistent obstacles, while smaller, well-defined transmission foci remain in Brazil and Venezuela. Caused by the filarial nematode *Onchocerca volvulus* and transmitted by blackflies, the disease causes progressive disability, economic loss, and hampers community development. Despite decades of mass drug administration with ivermectin, the inability of the drug to kill adult worms, increasing concerns over resistance, and the lack of effective vaccines continue to hinder elimination strategies. This review provides an overview of the disease, discussing its transmission dynamics, pathogenesis, clinical manifestations, diagnosis, and treatment. It also examines the immune-mediated responses that drive tissue damage and chronic morbidity, with an emphasis on skin and ocular complications. We highlight the need for rapid, non-invasive, and cost-effective diagnostic tools to enhance disease surveillance and assessment of treatment. Finally, we discuss the limitations of current therapies and recent progress in vaccine development, particularly subunit and multi-epitope vaccine candidates identified through reverse vaccinology approaches. Together, these insights reinforce the need for integrated strategies, combining improved diagnostics, novel treatments, and vaccines, to drive progress toward onchocerciasis elimination.

## 1 Introduction

Onchocerciasis, is a debilitating disease that is caused by *Onchocerca volvulus*, a nematode parasite (WHO, 2022). It affects millions of people in some parts of the world, particularly sub-Saharan Africa, presenting with severe dermatological and ocular complications that includes irreversible blindness (WHO, 2022). Beyond its health implications, onchocerciasis poses a significant barrier to socio-economic development in the affected regions. The disabling effect of the disease limits productivity by diminishing workforce participation and hindering learning by school children. The added burden on caregivers, along with stigma and social exclusion, exacerbates financial hardship. Together, these factors perpetuate the cycle of poverty and impair development in these regions.

The available treatment strategies against onchocerciasis have relied on mass drug administration with ivermectin, which reduces the microfilarial load and provides relief from some symptoms (Chippaux et al., 1995; Ndyomugyenyi et al., 2004; Remme, 2004). However, reports on emerging resistance combined with the drug’s inability to kill adult worms or ensure long-term transmission interruption pose a significant concern (Remme, 2004; Gebrezgabiher et al., 2019). Furthermore, severe adverse reactions including coma, encephalitis and even death have been reported in patients co-infected with *Loa loa* (Gardon et al., 1997; Boussinesq et al., 1998; Twum-Danso and Meredith, 2003). Additionally, the absence of an effective vaccine is a core problem for eradication efforts and underscores an urgent need to develop a newer and more effective anthelmintic. Therefore, attention has been directed towards elucidating the mechanisms of action of various compounds with anthelmintic or anti-Wolbachia properties. This review seeks to provide a comprehensive overview of onchocerciasis, highlighting its transmission dynamics, pathogenesis, clinical manifestations, immune-mediated responses, diagnosis and treatment landscapes. By synthesizing recent findings, the review also addresses limitations of current treatment and highlights recent progress in vaccine research aimed at improving control and achieving elimination.

## 2 Onchocerciasis overview

### 2.1 History of onchocerciasis

Human onchocerciasis, a parasitic infection caused by members of the genus *Onchocerca* has persisted over decades despite efforts to control and eradicate the disease. The genus lends its name from two Greek words ‘onchos’ and ‘kerkos’ meaning ‘hook’ and ‘tail’ respectively and occurs in sub-Saharan Africa and some foci in central and South America (Brattig et al., 2021). *O. volvulus* is a member of the family Onchocercidae, with 34 described species affecting a variety of ungulate hosts (Lefoulon et al., 2017). While *O. volvulus* is the only species known to cause human onchocerciasis (Krueger et al., 2007; Lefoulon et al., 2017), several other species have been reported to cause zoonotic onchocerciasis in humans. These include *O. lupi* (first described in a wolf), *O. dewittei japonica* (of Japanese wild boars), *O. gutturosa* (of cattle), *O. cervicalis* (of horses), and *O. jakutensis* (of cervids) (Otranto et al., 2011; Lefoulon et al., 2017; Cambra-Pellejà et al., 2020). Nevertheless, these zoonotic cases are rare as only 40 cases have been documented in humans worldwide (Cambra-Pellejà et al., 2020).

According to later accounts, O’Neill first observed the *O. volvulus* microfilaria in Ghana in a case of “craw-craw” in 1875, Manson subsequently identified the microfilaria in 1890 and Leuckart described its morphology in 1983 (Burnham, 1998; Cox, 2002; Cupp et al., 2011; Brattig et al., 2021). Around the early 1900s, the link between onchocerciasis, blindness and the blackfly, *Simulium damnosum*, as the transmission vector, completed the discovery of its life cycle (Burnham, 1998; Cupp et al., 2011). The reports validated the historical connection established by the inhabitants of the Red Volta River in Ghana between the biting flies and the occurrences of skin lesions and blindness (Burnham, 1998). Advances in molecular techniques led to the description of the vector chromosomes (Vajime and Dunbar, 1975) and then the genomics of *O. volvulus* (Vajime and Dunbar, 1975; Choi et al., 2016; Cotton et al., 2016). Additionally, the rickettsia-like endobacteria of filaria, *Wolbachia*, were first reported as intracellular bacterial symbiont of *Onchocerca* by Kozek and Marroquin in 1977, and its genome was characterized in 2016 (Kozek and Marroquin, 1977; Cotton et al., 2016). It is known that *Wolbachia* is present in all developmental stages of *O. volvulus*, although, its symbiotic relationship is not fully understood (Tamarozzi et al., 2011; Albers et al., 2012; Bennuru et al., 2016). However, evidence suggests that Wolbachia is both a metabolic provider, supplying heam, riboflavin and nucleotide precurssors for *O. volvulus* as well as its immune modifying shield, driving a neutrophil-biased inflammation that diverts or delays eosinophil-dominated killing responses (Nfon et al., 2006; Darby et al., 2012). This relationship has been an important discovery in the history and approach to treatment of the disease.

### 2.2 Epidemiology and impact

Onchocerciasis is found predominantly in the poorest communities within the tropics, where the warm humid climate provides ideal conditions for the survival and reproduction of both *O. volvulus* and its vector (Cheke et al., 2015; Onifade et al., 2024). It is the world’s most frequent infectious cause of blindness, after trachoma (Boatin et al., 2024). About 21 million people are infected, of which 14.6 million have skin disease and 1.15 million are visually impaired (Marie and Petri, 2025). Over time and with increased intervention, onchocerciasis, once more prominent in the Americas, is now confined to Brazil and Venezuela. Onchocerciasis is a more significant problem in Africa and the Yemen (Schmidt et al., 2022; WHO, 2022). The WHO reported a global estimate of 246 million people living in 29 countries in Africa where interventions are needed to eliminate onchocerciasis, excluding populations in unmapped regions. The (WHO, 2018) report stated that approximately a total of 205 million people lived in endemic areas with 30,561 in the Americas. By 2019, this number increased to a total of 217.1 million with 33,746 in the Americas (WHO, 2019). In 2020, these figures rose even further to 240 million worldwide with 35, 228 in the Americas (WHO, 2020). There remains more to be done to combat the disease.

Onchocerciasis causes enormous suffering as it not only affects human health but also hampers socio-economic development. The long-term disability and impaired vision associated with the disease have caused a reduction in agricultural and economic productivity in impacted regions (Ubachukwu, 2006; Alonso, 2009; Anong et al., 2014; Frallonardo et al., 2022). Fertile lands have been depopulated due to fear of the disease, resulting in famine and economic losses (Frallonardo et al., 2022; Boatin et al., 2024). Also, when the primary income earner is blind, the financial burden intensifies for the affected families and the community’s overall workforce decreases (Ubachukwu, 2006). This intensifies suffering and misery, traps the affected communities in poverty and hinders regional development.

Additionally, the dermatological symptoms and associated stigma cause both considerable discomfort and social exclusion and discrimination, as individuals with visual skin infection often receive lower wages than their healthy counterparts (Brieger et al., 1998; Ubachukwu, 2006). This has aggravated the challenges for the infected individuals and their families. Even more unfortunate is the fact that women experience this stigma more than men, as it either reduces their marriage prospects, decreases their chances of marriage or cause marital instability (Ubachukwu, 2006). Stigmatization further limits social engagement and exacerbates mental health challenges and increases the risk of mental health disorders (Litt et al., 2012).

Moreover, educational performance in schools also declines due to the distraction caused by itching, leading to impaired academic achievements (Ubachukwu, 2006). Children experience disruptions in their education and in addition take on caregiving roles for blind family members (Litt et al., 2012). The resulting poverty leads to the inability to access or afford education (Litt et al., 2012). Overall, the health-related quality of life is negatively impacted for people with onchocerciasis compared to the healthy population (Otache et al., 2022).

Beyond these socioeconomic, psychosocial and educational consequences, the measurable health burden of onchocerciasis remains considerable. Zhu et al. (2024), reported an estimated global age-standardized disability-adjusted life years (DALYs) of 15.8 per 100,000 population (95% UI: 9.4, 23.9 per 100,000 population), reflecting a 40.8% decrease (95% UI: − 45.0, − 37.1%) between 1990 and 2021 (Zhu et al., 2024), However, these estimates do not incorporate several important and under-recognized contributors to morbidity, including onchocerciasis-associated epilepsy (OAE) and mental health disorders (Amaral and Colebunders, 2025). Preliminary studies have estimated that depressive illness in filariasis patients results in 5.09 million DALYs, with an added 229,537 DALYs attributed to caregivers (Ton et al., 2015). Furthermore, OAE is estimated to contribute about 128,000 years of life lived with disability (YLDs) in East and Central Africa, constituting approximately 13% of the total YLDs linked to onchocerciasis and 10% of epilepsy-related YLDs (Vinkeles Melchers et al., 2018). These findings indicate that the current global burden metrics likely underestimates the true multidimensional impact of onchocerciasis.

### 2.3 Life cycle of the parasite

**Figure 1:**
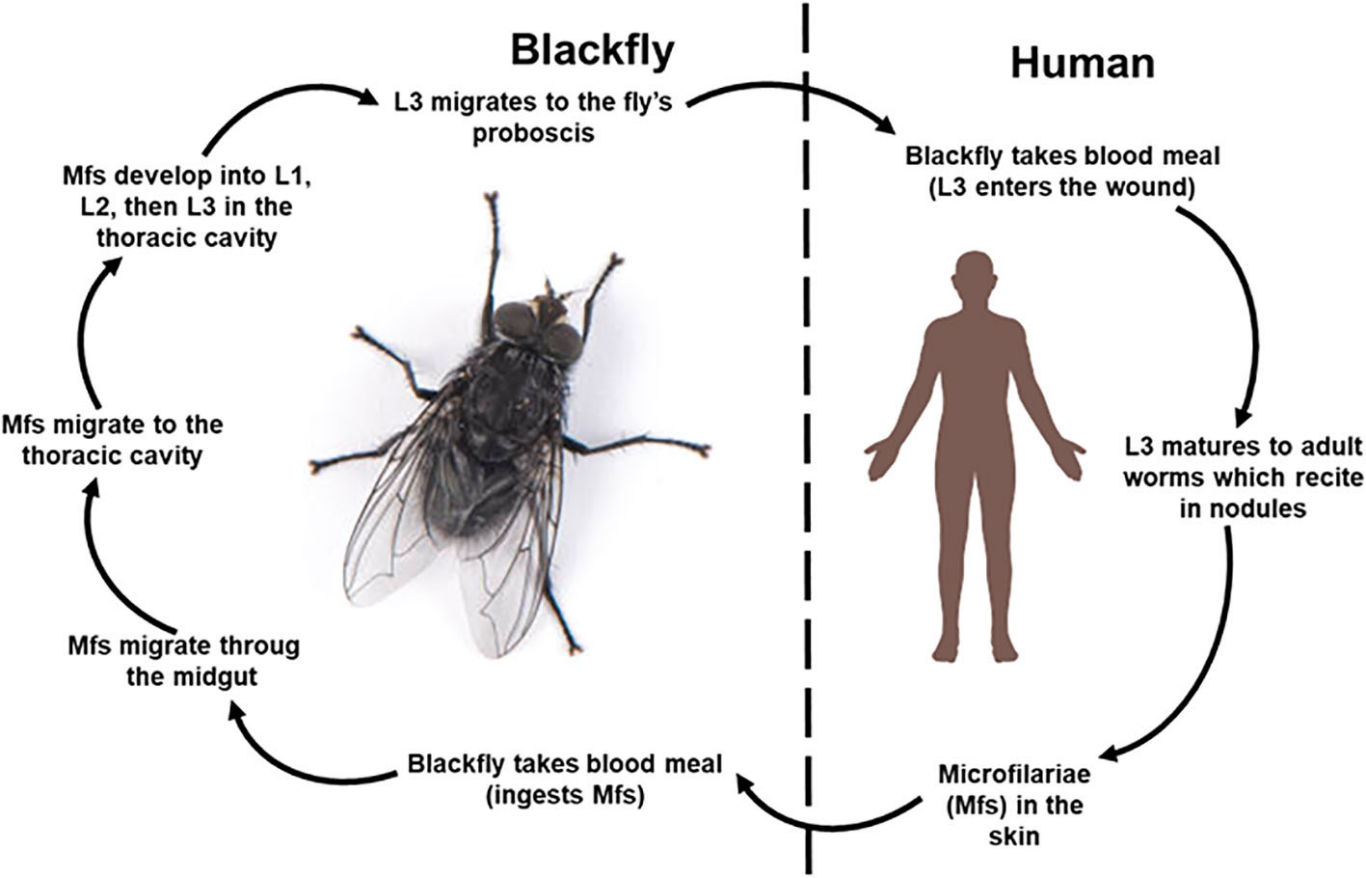
Life cycle of *Onchocerca volvulus.* The life cycle begins when the black fly takes a blood meal from an infected person and ingests microfilariae. The microfilariae migrate through the midgut to the thoracic cavity where they develop into the L1, and molt to the second stage larvae (L2) and finally the third stage infective larvae, L3. These L3 migrate to the fly’s proboscis and are transmitted to a human host during a subsequent meal. L3 matures into adults in human subcutaneous tissues. These adults reside in nodules (onchocercoma) where they mate, and females produce thousands of microfilariae daily. Human image and blackfly adapted from BioRender.com and https://www.istockphoto.com/photos/simulium-damnosum.

The life cycle (**Figure 1**) of *O. volvulus* is complex involving a human definitive host and an invertebrate intermediate host, the black fly, where all the larval stages exclusively develop (Gyasi et al., 2025). It begins when an infected black fly takes a blood meal and deposits the infective third-stage larvae (L3) into the skin of the human host. The larvae penetrates the skin through the wound and migrates to the subcutaneous tissues where they develop into adult worms over several months. While the developmental period is generally described as occurring within 6–12 months, studies from experimentally infected chimpanzees demonstrate that pre-patent period can extend up to 23 months, depending on the parasite strain (Duke, 1980; Nelson, 1991; Frallonardo et al., 2022). These adult worms live in painless fibrous nodules (onchocercomas) which typically measure between 0.5–3 cm in diameter and are commonly located near bony prominences such as the hips and ribs, but may also occur on the head and torso (Ali, 2006; Ajendra et al., 2022). Up to 50 aggregated female worms and around 10 males can be found within a nodule, distinguishing human onchocerciasis from other *Onchocerca* species, where female aggregation is not seen (Duerr et al., 2001; Brattig, 2004). Importantly, these nodules are not restricted to superficial locations but can also occur deep in tissues where they remain impalpable, despite occurring in the same anatomical regions as superficial nodules (Duke, 1980; Albiez, 1983; Kilian, 1988a). Such deep seated-nodules may not be detected during clinical palpation, underscoring the need for a more sensitive diagnostic approaches such as ultrasound-based nodule detection. While the aggregated females are sessile, males move from one nodule to another to inseminate the females (Duerr et al., 2001; Schultz-Key and Soboslay, 1994). The adult worms live for up to 15 years, and the females are generally longer and larger (33.5 to 50 cm x 270 to 400 μm) than the males (19 to 42 cm x 130-210 μm) (Lustigman et al., 2018; Dalton, 2021). Fertilized female worms develop microfilaria in 3 to 12 weeks, shedding 1300 to 1900 microfilariae daily for 9 to 11 years (Burnham, 1998). The microfilariae are immature, unsheathed larvae, 220 to 360 µm in length and 5–9 μm in diameter (Basáñez et al., 2006; Taylor and Pearlman, 2016). They migrate away from the adult and invade the skin, connective tissues and sometimes get to the eyes, and can live for up to 2 years within the host (Taylor et al., 2010; Dalton, 2021). The uninfected black flies ingest microfilaria from the skin which migrate from the midgut to the thoracic flight muscles (Dalton, 2021). Over a period of 1–3 weeks, the microfilaria molt into the first-stage larvae (L1), the second-stage larvae (L2), and finally, the infective third-stage larvae (L3) (Gyasi et al., 2025). These L3 larvae migrate to the fly’s proboscis, ready for transmission into a new host during the next blood meal, restarting the cycle (Voronin et al., 2019).

## 3 Pathogenesis

The pathology of onchocerciasis is mainly driven by the host immune response to the parasite, the parasite proteins and proteins form the *Wolbachia pipientis*, the bacteria endosymbiont. In *O. volvulus*, *Wolbachia* functions as an obligate mutualistic endosymbiont, residing not only throughout the parasite’s developmental stages (excluding the spermatocytes) but also within the reproductive tissues of female worms, where it is transmitted transovarially (Brattig, 2004). As a result, some pathological features associated with onchocerciasis are thought to arise from immune response to *Wolbachia* proteins released by dying microfilariae (Hotterbeekx et al., 2019). The accumulation of immune cells in response to infection eventually leads to chronic inflammation and tissue damage, which presents clinically as pruritis, rashes, depigmentation, nodules, skin atrophy, visual impairment and in severe cases, blindness.

### 3.1 Host immune response

The host immune response plays a pivotal role in both the development and progression of onchocerciasis. It involves a dynamic interaction between the innate and adaptive responses with different patterns in early versus chronic infection. In early infection, the immune system is more active, marked by elevated peripheral blood and mononuclear cell (PBMC) proliferation, granulocyte-macrophage colony stimulating factor (GM-CSF), IFN-γ (Th1) and IL-5 (Th2) responses, while chronic infection shows increased IL-10 and neutralizing antibodies (Elson et al., 1995; Turaga et al., 2000; Cooper et al., 2001; Kerepesi et al., 2005). Elevated IgG3 (GP20) levels in response to *O. volvulus* antigens are reported in individuals classified as “putatively immune” (PI) - those with high exposure to onchocerciasis but without nodules, microfilaremia or history of infection (Boyer et al., 1991; Stewart et al., 1995). Ward et al., 1988 also reported elevated IL-2 in PI individuals (Ward et al., 1988). This difference in immune response in putatively immune individuals compared to their infected counterparts suggests a protective immunity that prevents the establishment of an infection.

Conversely, in chronic infection elevated levels of IL-10 and TGF-β are present that mediate hypo responsiveness, suppressing Th1/Th2 activity and PMBC proliferation (Doetze et al., 2000; Lustigman et al., 2003). The antibody profile shifts and the non-cytophilic IgG4 becomes dominant, reducing antibody-dependent cellular cytotoxicity (Ottesen et al., 1985). This shift is favored by Treg cells and results in the promotion of tolerance (Satoguina et al., 2002; Johanns et al., 2022). The presence of circulating microfilariae in the blood and skin leads to elevated IgE levels and eosinophils (King and Nutman, 1991).

Persistent exposure to the parasite and on-going immune stimulation lead to nodule (onchocercoma) formation. The process involves the recruitment and infiltration of immune cells that are attracted by proteins released by these adult worms. Perivascular infiltration of leukocytes and tissue cells, such as fibroblasts and histiocytes, leads to granuloma formation, where macrophages differentiate into epithelioid cells and fuse to form multinucleated giant cells (Brattig, 2004). These nodules also harbor a diverse population of other immune cells including eosinophils, neutrophils, mast cells, T and B lymphocytes (plasma cells) and NK cells (Rubio de Krömer et al., 1998; Brattig, 2004). Nodule formation represents the host’s strategy to contain the parasite and limit tissue damage from the metabolic products it releases (Brattig, 2004). Overall, these immune responses contribute to inflammation, skin and eye lesions and the various clinical presentations observed in onchocerciasis. The severity of the infection is thus influenced by the host immunity and intensity of the parasite burden.

### 3.2 The role of Wolbachia

*Wolbachia* are gram-negative intracellular endosymbiotic bacteria found in *O. volvulus*, that play an important role in the parasite’s biology and host immune responses (Pietri et al., 2016; Hotterbeekx et al., 2019). When microfilariae die, they release *Wolbachia* and their protein components that trigger strong immune response such as inflammation and ocular lesions that may lead to blindness (Brattig, 2004; Idro et al., 2022). *Wolbachia* are essential for driving neutrophil-mediated inflammation, that contributes to keratitis, corneal opacity and haze (Gillette-Ferguson et al., 2004; Tamarozzi et al., 2011). They interact with neutrophils and stromal cells through the TLR2-MyD88 pathway, triggering CXC chemokine production and enhancing parasite inflammatory responses (Gillette-Ferguson et al., 2007; Tamarozzi et al., 2011). The observed reduction in neutrophil infiltration after *Wolbachia* elimination by doxycycline supports *Wolbachia’s* role in onchocerciasis pathology (Brattig et al., 2001). The mass release of *Wolbachia* following microfilaricidal treatment provokes, within a week systemic and skin-related side effects (Mazzotti reaction) including fever, lymphadenopathy, join pain, tachycardia, low blood pressure and pruritis (Tamarozzi et al., 2011). *Wolbachia* components also activate innate immune responses by interacting with Toll-like receptors TLR2 and TLR6 on host immune cells, particularly macrophages, exacerbating tissue damage and lesion formation (Hise et al., 2007). In chronic infection, *Wolbachia* lipoproteins stimulate IFN-γ, which indirectly enhances proinflammatory and chemotactic cytokine expression, thereby maintaining inflammation (Gentil and Pearlman, 2009; Turner et al., 2009; Tamarozzi et al., 2011). Through the stimulation of Neutrophil Extracellular Traps (NETs), *Wolbachia* also promotes the recruitment of immune cells at the sites of infection, contributing to nodule formation around parasites (Tamarozzi et al., 2016). Taken together, *Wolbachia* is a central mediator of immune-driven pathology in onchocerciasis. Beyond supporting the survival of *O. volvulus*, it significantly contributes to disease progression and clinical manifestations.

### 3.3 Tissue damage mechanisms

Tissue damage in onchocerciasis results are due to the cumulative effects of the host immune response to the dying microfilaria and their endosymbiont, *Wolbachia* (Ottesen, 1995; Frallonardo et al., 2022). The release of antigens from the dying microfilaria triggers both innate and adaptive immune responses, leading to immune cell recruitment, inflammation, and tissue damage (Brattig, 2004). Chronic inflammation causes dermatitis, depigmentation (leopard skin), lizard skin, scaring, loss of tissue elasticity, lymph node enlargement, keratitis, uveitis with ultimately, optic nerve damage and blindness (Thylefors, 1978; Taylor et al., 2010). These are further exacerbated by *Wolbachia*-driven pathology which is implicated in corneal opacity, keratitis, and nodule (onchocercoma) formation (Gillette-Ferguson et al., 2004; Hise et al., 2007). Additionally, long-term infection results in the development of a regulatory immune environment where there is Th1/Th2 cytokine imbalance that suppresses Th1 protective responses (Ali, 2006; Tamarozzi et al., 2016). This immunomodulation favors parasite persistence and survival, contributing to chronic inflammation and tissue damage. These processes play a central role in the clinical presentations of onchocerciasis. Thus, tissue damage in onchocerciasis causes cosmetic complications, while ocular involvement can lead to irreversible blindness.

## 4 Clinical manifestations

The clinical presentations of onchocerciasis typically appear years after the initial infection (Frallonardo et al., 2022). Adult worms live within subcutaneous nodules, and release large numbers of microfilariae that migrate through lymphatic pathways to skin, eyes, and sometimes the brain (Hotterbeekx et al., 2021a). The extensive migration and eventual death of microfilariae in the skin and ocular tissues trigger immunological and intense inflammatory reactions (Arndts et al., 2014; Frallonardo et al., 2022). Additionally, the nematode derived molecules also elicit immune responses (Brattig et al., 2000; Schönemeyer et al., 2001; Hotterbeekx et al., 2021a). These immune-mediated responses underlie the clinical manifestations of the disease, including chronic dermatitis, skin depigmentation, visual impairment and in severe cases, irreversible blindness (Ubachukwu, 2006). Clinically, individuals with onchocerciasis are categorized into three groups: those with generalized onchocerciasis (GEO), those with severe chronic dermatitis known as sowda, and a third group termed endemic normals (EN) (Tamarozzi et al., 2011; Arndts et al., 2014). The generalized form is marked by mild or intermittent skin symptoms despite high parasite loads, whereas sowda is characterized by a rare hyperreactive immune response resulting in severe skin lesions with a low parasite burden (Adjobimey and Hoerauf, 2010; Tamarozzi et al., 2011; Frallonardo et al., 2022). Also referred to as lichenified onchodermatitis, sowda typically presents with intensely itchy, hyperpigmented plaques and a darkened skin appearance (Murdoch, 2018; Lakwo et al., 2020). In contrast, endemic normals are individuals who have been exposed to the parasite for many years without developing any clinical symptoms (Brattig, 2004). These distinct clinical categories reflect the broader spectrum of onchocerciasis presentations, which primarily affect the skin (dermatological manifestations), eyes (ocular manifestations), and lymphatic system, with severity influenced by the host’s immune response.

### 4.1 Dermatological presentation

**Figure 2:**
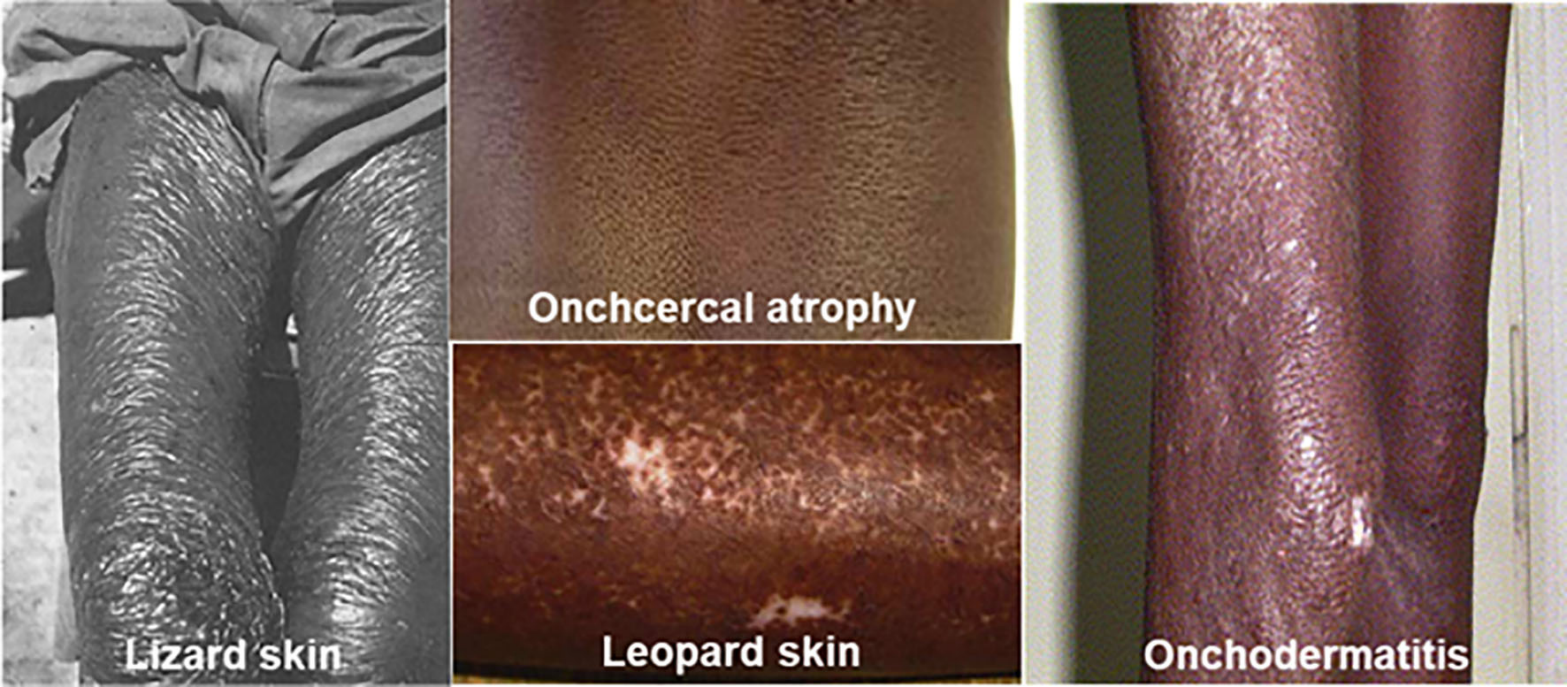
Skin manifestations of onchocerciasis (adapted from Stingl, P. (1997) and Enk, C. D. (2006)). Some visible signs of onchocerciasis including lizard skin (dry scaly skin), onchocercal atrophy, leopard skin (skin depigmentation) and onchodermatitis.

Among the earliest most noticeable symptoms are dermatological changes (**Figure 2**), characterized by a variety of skin lesions. These symptoms typically appear years after the initial infection (Frallonardo et al., 2022), a delay likely due to the long lifespan of adult worms and the slow accumulation of microfilariae. Over time, the immune response to foreign proteins released by the parasite and its endosymbiont, *Wolbachia*, leads to the formation of palpable nodules (Brattig, 2004). The nodules are a consequence of immune cell infiltration and granuloma formation, and range in size from about 2 mm to over 6 cm (Edgeworth et al., 1993; Brattig, 2004). Subcutaneous nodules known as onchocercomas can appear over bony prominences anywhere on the skin (Okulicz et al., 2004); however, the location varies by region, typically appearing above the waist in Central America and below the waist in Central Africa, reflecting the biting preferences of the blackflies (Tamarozzi et al., 2011; Frallonardo et al., 2022).

Another skin manifestation is onchodermatitis, an itchy rash caused by the immune response to migrating and dying microfilariae (Hoerauf et al., 2008b; Arndts et al., 2014; Hotterbeekx et al., 2019). It typically starts as acute papular dermatitis and can progress to the chronic form known as sowda or lichenified dermatitis (Hotterbeekx et al., 2019). In lichenified dermatitis, there is severe pruritis with hyperpigmented papules, plaques and thickened skin due to repeated inflammation (Tamarozzi et al., 2011; Hotterbeekx et al., 2019; Chakraborty et al., 2024). Other chronic forms of dermatitis present as lizard skin, leopard skin and atrophy. “Lizard skin” is characterized by dry itchy and scaly skin resembling lizard scales, while “leopard skin” is marked by extensive depigmentation and dark follicular spots, most prominently on the calves (Enk, 2006; Rieder, 2015; Noormahomed et al., 2016; Melchers et al., 2021). Chronic inflammation can lead to skin atrophy, with dryness, wrinkling, and loss of dermal elastic fibers, giving the skin a cigarette-like, aged appearance (Noormahomed et al., 2016; Hotterbeekx et al., 2019). This is often seen on the sacrum, buttocks and thighs (Stingl, 1997). In older individuals, hanging groins (adenolymphoceles) may develop due to lymphatic obstruction and cigarette skin (Connor et al., 1985; Stingl, 1997; Hotterbeekx et al., 2019).

### 4.2 Ocular manifestations (river blindness)

Onchocercal blindness is the clinical manifestation from which the disease lends its common name. Visual impairment occurs when microfilaria migrate to the cornea, anterior and posterior regions of the eye, where they get trapped, die and elicit immune responses (Newland et al., 1991; Hall and Pearlman, 1999; Gillette-Ferguson et al., 2004; Tamarozzi et al., 2011). The dying microfilaria also release molecules from their bacterial endosymbiont, *Wolbachia*, which attract immune cells and trigger inflammation (Hotterbeekx et al., 2019). In the early stage of corneal infections, reversible lesions appear as fluffy, snowflake-like opacities, known as “onchocercal punctuate keratitis” (Thylefors, 1978). Over time, the recurring inflammation and/or massive invasion of the cornea leads to chronic keratitis and sclerosing keratitis, gradually reducing corneal transparency and peripheral vision (Thylefors, 1978; Hotterbeekx et al., 2019). As opacification worsens, it culminates in total, irreversible vision loss (Hall and Pearlman, 1999). Infection in the anterior section causes uveitis, which can lead to complications like iris atrophy, obstruction of the iridocorneal synechiae, cataract and glaucoma (Thylefors, 1978; Tamarozzi et al., 2011). The presence of large numbers of microfilaria in the anterior section is likely a precursor to posterior involvement (Thylefors, 1978). Atrophy of the retinal pigment epithelium occurs, progressing to subretinal fibrosis in advanced lesions in posterior infection (Semba et al., 1990; Hall and Pearlman, 1999). Additionally, choroid retinal scarring, and post-neuritic optic atrophy may occur (Enk, 2006; Tamarozzi et al., 2011).

### 4.3 Neurological involvement

Neurological manifestations of onchocerciasis have been linked to neurological conditions such as epilepsy, acute cerebral ischemic/perfusion injuries, nodding syndrome and the Nakalanga syndrome (Bhalla et al., 2013; Berkowitz et al., 2015; Hotterbeekx et al., 2019). However, how *O. volvulus* triggers some of these manifestations remains unclear (Ndondo et al., 2022).

Epilepsy is a neurological disorder characterized by a repeated tendency to experience seizures without an obvious trigger (Scharfman, 2007). Although direct evidence is limited, a strong association has been suggested between epilepsy and onchocerciasis due to the high prevalence of epilepsy in onchocerciasis-endemic regions (Colebunders et al., 2018). This association is supported by several studies and meta-analyses (Pion et al., 2009; Kaiser et al., 2011, 2013; Siewe Fodjo et al., 2020), including those in Uganda and Cameroon, where the prevalence of epilepsy were significantly higher in onchocerciasis affected areas (Ovuga et al., 1992; Kaiser et al., 2011; Chesnais et al., 2018). Further evidence comes from observations in South Sudan, the Democratic Republic of Congo, and Tanzania, where seizure frequency was reduced after treatment with ivermectin in individuals with epilepsy and concurrent *O. volvulus* infection (Dusabimana et al., 2021). It is believed that the reduction is due to the action of ivermectin on the microfilariae rather that an effect of ivermectin on the host brain because ivermectin is readily excluded from by P-glycoprotein in blood-brain barriers of healthy individual (Schinkel et al., 1994; Geyer et al., 2009; Dusabimana et al., 2021). These observations led to the use of the term *Onchocerca* associated epilepsy (OAE) to describe such disorders and the concept of “river epilepsy” for epilepsy linked to onchocerciasis (Pion et al., 2009; Colebunders et al., 2019; Idro et al., 2022; Widdess-Walsh, 2024). What distinguishes OAE from other forms is its onset between ages 3 to 18, its occurrence in regions with high *O. volvulus* transmission, and the tendency to affect multiple siblings within affected families (Levick et al., 2017; Hotterbeekx et al., 2019).

The Nakalanga syndrome was first observed in 1950, in pigmies of the Banakalanga tribe in the Mabira forest Uganda, where onchocerciasis was endemic (Raper and Ladkin, 1950). Later on, similar cases were reported in other parts of Uganda, as well as sub-Saharan Africa, Burundi and Ethiopia (Föger et al., 2017). The condition typically affects children between 3 to 18 years of age, who had previously been developing normally (Ndondo et al., 2022). It is characterized by growth retardation, absence of secondary sexual characteristics, skeletal deformities, dental caries, and intellectual disability, sometimes with or without epileptic seizures (Kipp et al., 1996; Föger et al., 2017). The association between Nakalanga syndrome and onchocerciasis is strongly supported by both its presence in endemic areas and the absence of new cases with the implementation of onchocerciasis control programs in regions including the Mabira Forest in Uganda (Katabarwa et al., 2018; Hotterbeekx et al., 2019).

Nodding syndrome, another neurological disorder associated with onchocerciasis, is characterized by forward movement of the head due to loss of neck muscle tonus (Winkler et al., 2008; Hotterbeekx et al., 2019). These episodes are sometimes accompanied by a temporary loss of muscle tone in the upper extremities (Winkler et al., 2008). First described in Tanzania in the 1960s, it was subsequently reported in Uganda, Liberia, South Sudan, Cameroon, and the Democratic Republic of Condo (Idro et al., 2013; Föger et al., 2017). The Nodding syndrome is considered a distinct form of epilepsy that affects previously healthy children between 3 and 18 years of age, most of whom are infected with *O. volvulus.* (Winkler et al., 2008; Dowell et al., 2013; Ogwang et al., 2021; Ndondo et al., 2022). Clinical symptoms include atonic seizures, psychiatric symptoms, cognitive decline, impaired growth and delayed puberty (Dowell et al., 2013; Idro et al., 2013).

The remarkable similarity in age of onset, developmental retardation, and neurological symptoms has led to speculation that Nakalanga and Nodding syndromes may be two clinical expressions of the same underlying pathology linked to onchocerciasis (Föger et al., 2017).

The link between onchocerciasis and neurological syndromes has prompted several, sometimes conflicting, hypotheses, although definitive causal mechanisms remain unproven. An earlier study proposed that cross-reactivity between antibodies to human leiomodin-1 and an *O. volvulus* antigens could underlie neuronal injury in Nodding syndrome, supporting a post-infectious autoimmune mechanism (Johnson et al., 2017). However, subsequent studies found no difference in leiomodin-1 antibody prevalence between Nodding/OAE cases and controls, challenging the role of this autoantibody as a primary pathogenic driver (Hotterbeekx et al., 2021b). A further emerging hypothesis is that nematode-borne viruses could trigger pathology (Quek et al., 2024). Although omics evidence for a neurotropic virus in *O. volvulus* worms is absent, studies demonstrated an abundance of OVRV1, an RNA virus in adult reproductive tissues that elicits antibody responses indicating direct exposure to the immune system (Colebunders et al., 2023; Quek et al., 2024). Additionally, studies indicate a dose-dependent relationship between microfilarial load and epilepsy risk in children, supporting the proposition that high-intensity infection drives neuroinflammatory processes leading to seizures (Hadermann et al., 2023). Accordingly, improved onchocerciasis control may reduce the incidence of related neurological syndromes.

## 5 Diagnosis

Accurate diagnosis of onchocerciasis is essential for effective control, patient management, epidemiological mapping and success of monitoring and elimination programs. In endemic regions, presumptive diagnosis of onchocerciasis is primarily based on physical examination, focusing on identifying characteristic dermatological and ocular manifestations. However, this approach is limited in that it cannot reliably differentiate onchocerciasis from other infections with similar clinical presentations. To address this, definitive diagnostic methods were developed that can confirm the presence of *O. volvulus* and improve diagnostic accuracy (Bradley and Unnasch, 1996; Boatin et al., 1998). Over time, these approaches have evolved from the traditional parasitological methods to more advanced serological and molecular techniques, each presenting unique benefits and challenges.

### 5.1 Parasitological diagnosis

Traditionally, the skin snip microscopy has served as the gold standard and most widely used method for diagnosing active onchocerciasis infection (Hotterbeekx et al., 2020). It involves collecting superficial skin biopsies, typically from the scapula, iliac crest, or calf, incubating it in saline for 30 minutes or longer to allow for the emergence of microfilaria for microscopic examination (Kale et al., 1974; Vincent et al., 2000; Vlaminck et al., 2015; Frallonardo et al., 2022). Although this method is very specific, its sensitivity remains low in early infection, hypo endemic areas or in individuals with low microfilarial density especially after treatment with ivermectin (Vincent et al., 2000; Boatin et al., 2002; Thiele et al., 2016). Additionally, skin snip is invasive and painful, requiring expensive equipment and proper sterilization between subjects and carries a risk of blood-borne pathogen transmission (Vincent et al., 2000; Boatin et al., 2002). Given these drawbacks, the WHO recommended against using the skin snip method as a primary diagnosis to verify elimination (Unnasch et al., 2018). A non-invasive method of diagnosis is nodule palpation, which detects subcutaneous onchocercomas (Frallonardo et al., 2022). However, it lacks sensitivity and specificity, particularly in early or low intensity infections, often resulting in missed cases and underestimation of infection prevalence.

### 5.2 Serological diagnosis

To overcome the limitations associated with parasitological diagnostic techniques, serological tests, particularly those specific for the detection of IgG4 antibodies against the *O. volvulus* OV16 antigen have been used (Brattig et al., 2021). They are available as both Enzyme-linked immunosorbent assays (ELISA) and rapid diagnostic tests (RDTs) and have been particularly useful for children and for onchocerciasis elimination mapping (Weil et al., 2000; Golden et al., 2016; Albert et al., 2021; Shintouo et al., 2021). In addition to the OV16 antigen, several alternative and complementary biomarkers including OVOC10469, OVOC3261 and OvMANE1 have been explored to enhance the sensitivity and specificity, while OvHSP70 has offered a urine-based diagnostic alternative (Bennuru et al., 2020; Shintouo et al., 2021; Ambe et al., 2024). Serological techniques provide less invasive alternatives to skin snips and are suitable for large-scale mapping and monitoring. However, they still pose a limitation in that they cannot distinguish between past and current infection and require infrastructure that is not always available in endemic areas (Shintouo et al., 2021; Frallonardo et al., 2022). Additionally, cross reactivity presents a significant limitation in the serological diagnosis of onchocerciasis, particularly in regions where multiple filarial infections coexist (Bartlett et al., 1975; Lagatie et al., 2019).

### 5.3 Molecular diagnosis

Molecular techniques such as polymerase chain reaction (PCR) and the loop-mediated isothermal amplification (LAMP) provide greater specificity and sensitivity in detecting the *O. volvulus* DNA in skin snip, compared to microscopy (Tang et al., 2010; Lagatie et al., 2016). The *cox*1 LAMP for example, provides results within 30 minutes and performs comparably to the O-150qPCR (Lagatie et al., 2016). These techniques distinguish between different filarial species, hence overcoming the problem of cross-reactivity in serological techniques. Their high sensitivity makes them suitable for detecting infections in regions with low or decreasing prevalence after ivermectin treatment (Lloyd et al., 2015; Amambo et al., 2023). However, the high cost of PCR remains a barrier to its routine use in epidemiological surveillance (Moya et al., 2016).

### 5.4 Indirect diagnosis

The Mazzotti test was first introduced in the 1940s as an indirect diagnostic test to detect *O. volvulus* (Tada et al., 1981). It is based on the host’s clinical reaction to diethylcarbamazine (DEC), an anti-filarial drug. In this test, a low dose of oral or topical DEC is administered, triggering a papular, pruritic rash within 24 hours in patients with active microfilariae (Stingl et al., 1984; Kilian, 1988b; Daniel-Nwosu et al., 2021). This reaction is due to the rapid killing of the microfilariae by the drug eliciting host immune response. The reaction is also seen in patients treated with ivermectin (Gyasi et al., 2025). While the Mazzotti test was historically valuable for identifying active onchocerciasis, its lack of specificity and the severity of potential side effects have led to a decline in its use.

Future progress will depend on the development and deployment of rapid, reliable, cost-effective, and non-invasive diagnostics to enhance disease surveillance, monitor treatment outcomes, and support elimination efforts.

## 6 Treatment

The control of onchocerciasis can be broadly classified as non-pharmacological and pharmacological, with the latter being the most heavily used. Due to the absence of an effective vaccine, the disease management has relied primarily on therapeutic interventions. However, a major barrier to therapeutic strategies has been the absence of an ideal drug that kills both the adult worm (macrofilaricidal) and its microfilariae (microfilaricidal) with minimal side effects in patients. Over the past decades, treatment strategies have advanced from individual treatments and symptom management to large-scale community-based mass drug administration (MDA) programs aimed at interrupting transmission. In this section we discuss these strategies, their inherent limitations and drug resistance in onchocerciasis.

### 6.1 A non-pharmacological approach

#### 6.1.1 Nodulectomy

Early before the advent of drugs, treatment of onchocerciasis was by nodulectomy, a procedure that required surgical removal of the nodules (Richards et al., 2015). However, results for this procedure were only positive for hypo endemic areas as new nodules developed rapidly for patients in hyperendemic areas (Albiez, 1985; Guderian, 1988). Additionally, nodulectomy did not significantly reduce microfilarial density in patients with established infections in the rain-forest zone of Western Nigeria (Kale, 1982). Thus, the procedure is not suitable for patients with multiple, deep seated, or non-palpable nodules. Additionally, it does not address microfilariae responsible for clinical symptoms. As a result, nodulectomy can only be performed occasionally as the need arises.

### 6.2 Pharmacological approaches

#### 6.2.1 Individual-level treatment

Treating onchocerciasis at the individual-level has involved both pharmacological and non-pharmacological strategies, with the primary goal of alleviating symptoms, reducing disease burden and progression. This section explores historical and therapies under investigation.

##### 6.2.1.1 Suramin

Suramin, introduced in 1916, was the first drug used against onchocerciasis and was found to be both macrofilaricidal and microfilaricidal (Burch and Ashburn, 1951; Duke, 1968b). Suramin acts by inhibiting dihydrofolate reductase in *O. volvulus* (Gupta and Srivastava, 2005), disrupting its folate metabolism and ultimately impairing DNA synthesis and cell proliferation. The drug was used against onchocerciasis until the 1970s, but later discontinued due to its severe toxicity (Higazi et al., 2014). Observed adverse reactions included skin reactions (“suramin casts”), idiosyncratic reactions (such as nausea, vomiting, loss of consciousness), and even death (Anderson et al., 1976; Awadzi, 2003; Higazi et al., 2014; Tagboto and Orish, 2022). Although no longer used for onchocerciasis, suramin continues to be used primarily for stage 1 trypanosomiasis, as suramin does not cross the blood-brain barrier (Bouteille and Buguet, 2012; Babokhov et al., 2013).

##### 6.2.1.2 Diethylcarbamazine

Discovered in 1947, diethylcarbamazine, a derivative of piperazine was administered and showed microfilaricidal activity but was not effective in killing the adult *Onchocerca* worm (Duke, 1968b; Subrahmanyam, 1987; Robertson et al., 2012). The microfilaricidal action of diethylcarbamazine (DEC) was earlier believed to be indirect, functioning through the inhibition of arachidonic acid metabolism, thereby modulating the host’s inflammatory response (Maizels and Denham, 1992; McGarry et al., 2005; Robertson et al., 2012). However, later studies showed that DEC targets TRP-2 channels in the worms causing subsequent activation of SLO-1K channels and eventual paralysis (Verma et al., 2020). Unfortunately, its tendency to provoke severe adverse effects (Mazzotti reaction) especially in patients with high microfilariae load posed a major limitation to its use (Greene et al., 1983; Gyapong and Boatin, 2024). As a result, DEC is contraindicated and not used as a primary strategy for the treatment of onchocerciasis (Tagboto and Orish, 2022). However, it is being re-examined as a triple therapy with ivermectin and albendazole and is currently used for diagnostic purposes (Ozoh et al., 2007). Additionally, although larger trials are needed, research suggests that DEC-fortified salt is safe, effective, and well tolerated in endemic areas, implying that delivery strategies may mitigate adverse effects (Nwaiwu et al., 2022).

##### 6.2.1.3 Melarsonyl potassium

As drug discovery advanced, melarsonyl potassium, an arsenical, was developed in 1949 and was found to be effective against adult *O. volvulus* but not its microfilaria (Duke, 1970; Tagboto and Orish, 2022). The arsenicals act by (i) disrupting glucose uptake and metabolism (ii) inhibiting glutathione reductase and (iii) altering the structure and function of the surface of intestinal epithelium of parasites (Subrahmanyam, 1987). However, it was discontinued due to associate risk of inducing encephalopathy leading to death (Duke, 1966; Tagboto and Orish, 2022). Nevertheless, it is still used to treat certain forms of trypanosomiasis, though efforts are underway to develop safer and more convenient alternatives (Lindner et al., 2025).

##### 6.2.1.4 Doxycycline

Doxycycline, a second-generation, semi-synthetic tetracycline antibiotic developed in the early 1960s by Pfizer as a broad-spectrum antimicrobial (Tan et al., 2011). It was introduced under the trade name Vibramycin, approved by FDA in 1967, and was used to treat bacterial infections respiratory, and sexually transmitted diseases (Tan et al., 2011; Tiwaskar, 2024). Its unique ability to target *Wolbachia*, the bacterial endosymbionts essential for *O. volvulus* survival, led to its use to treat onchocerciasis. Doxycycline binds to the bacterial ribosomal subunit, inhibiting protein synthesis (Tiwaskar, 2024). By targeting *Wolbachia* it inhibits embryogenesis thereby sterilizing the female worms and indirectly resulting in microfilaricidal activity (Debrah et al., 2015). Clinical studies have demonstrated that six-week doxycycline course blocks embryogenesis and reduces microfilariae for over a year without serious side effects (Hoerauf et al., 2003; Turner et al., 2010). Doxycycline is also valuable in regions co-endemic with *Loa loa* given the absence of *Wolbachia* in this species (Büttner et al., 2003; McGarry et al., 2003; Turner et al., 2010). However, the long treatment duration, and contraindications seen in pregnant women and children <8 years old pose significant challenges to its use, particularly in MDA programs; nevertheless, it may be well suited for a test-and-treat intervention (Tagboto and Orish, 2022).

##### 6.2.1.5 Levamisole

Levamisole discovered in 1966, acts on the nicotinic acetylcholine receptor at the neuromuscular junction, causing sustained muscle contraction and eventual paralysis of the worm (Janssen, 1976; Robertson and Martin, 1993; Robertson et al., 1999; Campillo et al., 2022b). It has been successfully used to treat ascaris and *Trichuris trichiura* and hookworm infections (Horton, 2003; Campillo et al., 2022b). Given this broad spectrum of activity, levamisole was considered for the treatment of onchocerciasis. In particular, its reported effectiveness against equine onchocerciasis in Southwestern British Colombia (Lees et al., 1983) supported further investigations and trials in humans. However, although animal model studies revealed some microfilaricidal activity against *O. lienalis*, its effect on the motility of *O. gutturosa* adult worms was inconsistent (Townson et al., 1987, 1988). Clinical trials in humans showed promise for *Loa loa* but not for onchocerciasis as levamisole failed to demonstrate significant microfilaricidal or microfilaricidal activity (Rivas-Alcalá et al., 1981; Awadzi et al., 2004; Campillo et al., 2022a). Consequently, its development for onchocerciasis was abandoned.

##### 6.2.1.6 Auranofin

First synthesized in 1972 by Surron et al., auranofin was developed as a therapeutic agent for rheumatoid arthritis and FDA approved in 1985 (Sutton et al., 1972; Capparelli et al., 2016). The drug acts by inhibiting thioredoxin reductase, a critical enzyme responsible for mitigating oxidative stress and cell damage (Rigobello et al., 2004). Auranofin’s efficacy against *Brugia* adult worms in gerbil also supports thioredoxin reductase enzyme as a likely target in nematodes (Bulman et al., 2015). Although auranofin was effective in inhibiting the molting of *O. volvulus* L3s, and killing both pre-adult and adult *O. volvulus* worms *in vitro* (Bulman et al., 2015; Voronin et al., 2019), there is no direct evidence of its efficacy in humans. Results from a phase I trial showed that auranofin was safe when orally administered to healthy individuals (Capparelli et al., 2016). However, no clinical trials have been initiated to assess the efficacy of auranofin against human onchocerciasis. Consequently, the lack of evidence of its effectiveness and safety in infected individuals has hindered further development. Attention has since shifted to other candidates with stronger potential for clinical translation.

##### 6.2.1.7 Amocarzine

Investigated in the late 90s, amocarzine also known as (CGP 6140), showed both microfilaricidal and macrofilaricidal activity against *O. volvulus* (Poltera et al., 1991; Tagboto and Orish, 2022). It does not belong to any class of known anthelminthics and its mode of action has not been fully investigated (Poltera, 1994). It was suggested that amocarzine inhibits mitochondrial function and acetylcholinesterase activity (Köhler et al., 1992). Although amocarzine was shown to be effective when administered at low doses (Guderian et al., 1991), results from different studies were not consistent. While it was found to be both microfilaricidal and macrofilaricidal in studies in Guatemala, the effect was only microfilaricidal in trials done in Ghana (Tagboto and Orish, 2022). Additionally, an early study by Awadzi et al. (1997) demonstrated that amocarzine had no role in onchocerciasis treatment (Awadzi et al., 1997). This together with the severity of the Mazzotti-type reactions observed with amocarzine disfavored its use for onchocerciasis treatment (Tagboto and Orish, 2022).

##### 6.2.1.8 Benzimidazoles

The benzimidazoles, including mebendazole, albendazole and flubendazole that came into clinical use in the 1990s (Mahurkar et al., 2023), have also been investigated for onchocerciasis treatment. They act by inhibiting microtubule formation within the parasite resulting in the disruption of critical functions associated with microtubes such as cell division, cell motility, nutrient absorption among others (Lacey, 1990; Martin, 1997; Claerebout et al., 2025). The benzimidazoles demonstrated significant differences in their stage-specific efficacy. Mebendazole showed microfilaricidal activity and selectively targeted developing embryos within eggshells but not the stretched microfilariae (Awadzi, 1997). On the other hand, albendazole was effective against intrauterine stages but lacked microfilaricidal activity (Cline et al., 1992; Awadzi, 1997). The lack of macrofilaricidal activity of mebendazole and albendazole limited their use in onchocerciasis treatment.

Flubendazole, another derivative of benzimidazole approved for the treatment of human gastrointestinal nematode infections, demonstrated macrofilaricidal activity in a double-blind clinical study in human (Dominguez-Vazquez et al., 1983). In this study, injectable formulations induced slow reduction in skin microfilaria load, over a period of 12 months. However, subsequent investigations demonstrated that oral formulations failed to achieve comparable efficacy, even when bioavailability was enhanced through multiple oral dosing regimens (Fischer et al., 2019; Sjoberg et al., 2019). Additionally, flubendazole was found to exhibit toxicity in preclinical studies (Lachau-Durand et al., 2019). Collectively, these limitations, together with the undesirable reactions observed at injection sites, curtailed further clinical development even though reports suggested a more favorable safety profile compared with DEC (Dominguez-Vazquez et al., 1983; Higazi et al., 2014; Geary et al., 2019; Lachau-Durand et al., 2019). Additionally, a preclinical toxicity study with formulations that yield improved systemic absorption reported in toxic reactions further hindering its development for onchocerciasis (Lachau-Durand et al., 2019).

##### 6.2.1.9 Antimonial compounds

Antimonial compounds used for the treatment of leishmaniasis and schistosomiasis (Martin and Lee, 2025), were among the earliest drugs evaluated for the treatment of onchocerciasis. Yet, none of them advanced for onchocerciasis therapy for reasons including lack of significant activity and or toxicity concerns. Their mode of action is multifaceted and includes inhibition of trypanothione reductase and type I DNA topoisomerase, enzymes involved in oxidative stress defense and DNA replication respectively in *Leishmania* spp (Chakraborty and Majumder, 1988; Wyllie et al., 2004). In schistosomes, they inhibit phosphofructokinase, a glycolytic enzyme and thioredoxin glutathione reductase, which maintains redox balance (Mansour and Bueding, 1954; Kuntz et al., 2007). Antimony compounds were shown to inhibit glycolysis in filariids *(Litomosoides carinii, Dipetalonema witei* and *Brugia pahangi)*, encouraging evaluation for onchocerciasis (Saz and Dunbar, 1975; Baiocco et al., 2009). Culbertson assessed seven of these antimony compounds namely; neostibosan, neostam, urea stibamine, stibanose (stibogluconate), fouadin (stibophen), anthiomaline and tartar emetic against Bancroftian filariasis (Culbertson, 1948). He reported severe reactions with the various drugs, except for neostibosan, which was well tolerated and effective. However, despite the efficacy of neostibosan, the extended follow-up period of over 26 months reflected its slow action and limited its value for therapeutic advancement. Contrary to his findings, Bartter et al. (1948) observed no severe reactions with stibophen and tartar emetic in volunteers with onchocerciasis (Bartter et al., 1948). However, the compounds did not show any significant reduction in the numbers of microfilariae or adult worm motility post-treatment, again limiting its therapeutic potential. Satti and Kirk (1957) evaluated pentostam and although he reported no toxicity, the compound lacked efficacy at the tested doses (Satti and Kirk, 1957). Duke (1968a) also evaluated stibocaptate (TWSb) and Friedheim (MSbE) and reported some microfilaricidal and macrofilaricidal or sterilization effects on *O. volvulus* adult worms, but noted toxic reactions that limited their therapeutic relevance (Duke, 1968a). Consequently, none of these antimony compounds progressed for development against onchocerciasis.

##### 6.2.1.10 Other drugs

Several other drugs have been evaluated for onchocerciasis, but their development was discontinued either due to limited microfilaricidal or macrofilaricidal activity or due to toxicity concerns. Pyrantel pamoate for instance, a nicotinic acetylcholine receptor agonist (Robertson et al., 1994), fully inhibited mobility in *O. gutturosa* male worms but lacked significant activity against *O. volvulus* microfilariae and adults (Kale, 1978; Gokool et al., 2024). Closantel, another veterinary anthelmintic, a chitinase inhibitor was found to inhibit molting of *O. volvulus* L3 larval stage (Gloeckner et al., 2010). However it was also reported to be toxic, causing blindness in humans (Essabar et al., 2014; Tabatabaei et al., 2016).

Anti-Wolbachia agents have also been explored for their potential to sterilize and eventually kill adult worms. While some candidates have advanced into clinical trials including Phase II, none have yet progressed to routine use in MDA programs. For instance, AWZ1066S, a synthetic azaquinazoline molecule demonstrated excellent preclinical results, but further development was stopped due to adverse events observed in Phase I trials (Hong et al., 2019; Devereux et al., 2024). Flubentylosin, a semisynthetic macrolide derived from tylosin A was safe and well tolerated in Phase I trials, but its development was discontinued following unfavorable efficacy results in Phase II (Dällenbach, 2015; Alami et al., 2023). Furthermore, some antibiotics with anti-*Wolbachia* activity have also been studied. Minocycline, a tetracycline antibiotic, has been shown to effectively deplete *Wolbachia* and block embryogenesis (Sharma et al., 2016; Klarmann-Schulz et al., 2017). However, its embryostatic and curative potential have not been confirmed in a Phase II trial (Klarmann-Schulz et al., 2017). Rifampin, an anti-tuberculosis drug, derived from rifamycin B, showed efficacy against *O. lienalis* microfilariae at 100 mg/kg given daily for 15 days in mice with comparable efficacy to a single 2 µg/kg dose of ivermectin (Townson et al., 2000; Campbell et al., 2001; Alsayyed, 2004). However, results from studies in *O. volvulus* were inconsistent. While Richards et al. (2007) found no effect, subsequent studies indicated that although low-dose rifampin is less potent than doxycycline, a high-dose short-course regimen shows superior efficacy (Specht et al., 2008; Aljayyoussi et al., 2017). However, because rifampin is a cornerstone first-line tuberculosis (TB) treatment population-level exposure in individuals with unrecognized latent or active TB could exert selective pressure for rifampin-resistant *Mycobacterium tuberculosis*, posing a risk to TB control efforts (Decroo et al., 2020; Cebrián-Sastre et al., 2023; Haigh et al., 2024).Still, concerns about toxicity at escalated doses hindered its progress for onchocerciasis treatment. Yet again, azithromycin, an azalide derived from erythromycin was trialed in Ghana, but it showed no efficacy against *Wolbachia* from *O. volvulus*, indicating that it is not suitable for onchocerciasis treatment (Richards et al., 2007; Hoerauf et al., 2008a; Bakheit et al., 2014). As a result, its development was halted.

#### 6.2.2 Community-based interventions

Community-based interventions are public health strategies that administer treatment or preventive measures to the entire population at risk. The aim of such interventions is to control or eliminate the disease by reaching entire communities especially where resources are limited, and individual treatment is not pragmatic. In the context of onchocerciasis community-based interventions have been offered through mass drug administration (MDA) with ivermectin or moxidectin and have been instrumental in interrupting transmission.

##### 6.2.2.1 Ivermectin

Ivermectin is an avermectin, a group of compounds that belong to the class of macrocyclic lactones. It was discovered in the late 1970s by a Japanese biochemist Satoshi Omura in collaboration with William Campbell, an Irish parasitologist whose work earned them a Nobel Prize (Tagboto and Orish, 2022). Following its remarkable success in veterinary medicine, ivermectin was described as a “wonder” drug with significant potential for human use (Crumb and Ōmura, 2011). It was rapidly advanced to human trials, where it demonstrated effective microfilaricidal activity and was approved for the treatment of human onchocerciasis in 1987 (Higazi et al., 2014). Compared to earlier treatments, ivermectin was safer and better tolerated, with milder and less severe side effects (Taylor and Greene, 1989). Its mechanism of action involves the activation of glutamate-gated chloride channels in the microfilariae, which impairs protein secretion necessary for immune evasion, ultimately leading to rapid parasite clearance (Moreno et al., 2010). A single dose significantly reduces microfilaria load, alleviating symptoms and preventing further transmission (Newland et al., 1988; Taylor, 1989). Ivermectin became the first anthelmintic to demonstrate feasible chemotherapy suitable for large scale treatment mass drug administration (Ali, 2006).

Although ivermectin is primarily microfilaricidal, studies have shown that repeated doses for extended periods have macrofilaricidal effects and can reduce female worm fecundity (Duke et al., 1990; Gardon et al., 2002; Cupp et al., 2011). However, the benefits are modest, and repeated or high dose of ivermectin increases the risk of toxicity (Duke et al., 1990; Yang, 2012; Gyasi et al., 2025). Ivermectin is also a substrate for P-glycoprotein, the multidrug resistant protein involved in drug distribution and elimination in organs such as the brain (Robertson et al., 2012; Njeshi et al., 2024). Defects in this transporter can result in neurological toxicity during ivermectin treatment. Although ivermectin continues to be the drug of choice for onchocerciasis treatment, its lack of effective macrofilaricidal activity limits its efficacy for elimination strategies. Ivermectin must be taken for about 10–15 years for infection to be cleared, which raises compliance issues that further hinders control efforts (Otabil et al., 2023; Gyasi et al., 2025). Additional concerns include emerging resistance and severe adverse reactions in patients co-infected with *Loa loa*, such as encephalopathy (Twum-Danso, 2003; Osei-Atweneboana et al., 2011; Chandler, 2018; Tagboto and Orish, 2022). These limitations emphasize the urgent need for improved therapeutic options.

##### 6.2.2.2 Moxidectin

Moxidectin is another semi synthetic macrocyclic lactone with superior microfilaricidal activity to ivermectin as it has a longer half-life (Tagboto and Orish, 2022). Initially developed for veterinary use, moxidectin’s potential for human onchocerciasis was recognized in the 1990s and approved by FDA in 2018 for onchocerciasis therapy (Prichard and Geary, 2019). Its safety profile is comparable to that of ivermectin, and no serious adverse effects have been reported, supporting its use for mass drug administration (Bjerum et al., 2023). However, given its increase potency, the Mazzotti reaction is more common compared to those with ivermectin treatment (Tagboto and Orish, 2022). Additionally, regulatory approval for children <12 years is still pending. These limitations again hinder its use for onchocerciasis treatment (Kura et al., 2023). Nevertheless, moxidectin has recently been introduced for a community-based MDA campaign for the first time in Ghana (Rugarabamu, 2025).

##### 6.2.2.3 Combination therapies

Drug combination therapies offer a valuable approach to improving therapeutic efficacy, minimizing adverse effects and delaying the emergence of resistance. To overcome the limitations of monotherapy, several drug combinations have been used for onchocerciasis treatment.

##### 6.2.2.4 Ivermectin combination therapies

Although combination therapy could enhance treatment outcomes, several studies have shown that combining ivermectin with other anthelmintics, offers no advantage over ivermectin monotherapy for onchocerciasis in terms of efficacy. They neither increase macrofilaricidal activity nor improve microfilariae clearance. However, these combinations are consistently reported to be beneficial in terms of safety and tolerability. For instance, ivermectin combined with albendazole was found to be safe but not macrofilaricidal, or superior to ivermectin (Awadzi et al., 2003; Olsen, 2007). This combination was also well tolerated in individuals coinfected with *Wuchereria bancrofti*, although it did not reduce microfileraemia (Makunde et al., 2003). The triple therapy of ivermectin, diethylcarbamazine and albendazole (abbreviated as IDA) has been studied for lymphatic filariasis (LF); however, its use for onchocerciasis remains limited and controversial due to risk of severe adverse reactions with diethylcarbamazine in onchocerciasis patients (King et al., 2018; Edi et al., 2019; Weil et al., 2019; Njenga et al., 2024). To address this, Fischer et al. (2019) proposed a “pretreat and treat” strategy, in which a microfilaria-clearing dose of ivermectin will be administered first, followed several months later by IDA. By pretreating with ivermectin, the microfilarial burden is reduced to safer levels, lowering the risk of severe adverse effects from diethylcarbamazine. Subsequent treatment with IDA will be safer and offer more effective long-term clearance of *O. volvulus* microfilariae than ivermectin monotherapy. However, risks to these strategies include residual microfilariae that could still trigger side effects, complex organization, uncertain long-term safety and the potential for resistance to develop.

Other studies have combined ivermectin and doxycycline to leverage the rapid microfilaricidal activity of ivermectin alongside the indirect macrofilaricidal effects of doxycycline. Although improvement in clinical symptoms have been reported, interpretation of ocular pathology is challenging as such outcomes require prolonged follow-up and are difficult to assess in settings with ongoing transmission (Masud et al., 2009; Abegunde et al., 2016). Consequently, most trials have assessed parasitological efficacy by comparing doxycycline plus ivermectin with ivermectin alone to determine the added benefit of doxycycline. Unfortunately, this approach limits direct evaluation against doxycycline monotherapy. Nevertheless, one randomized controlled trial assessed doxycycline plus ivermectin versus doxycycline alone and reported amicrofilaridermia in 89% and 67% of participants respectively at 21 months (Turner et al., 2010). However, this difference was only marginal and not statistically significant.

Ivermectin combined with levamisole, suramin, mefloquine, mebendazole and flubendazole against *Onchocerca* spp showed no therapeutic advantage over ivermectin alone (Townson et al., 1990; Cross et al., 1997; Awadzi et al., 2004; Tagboto and Orish, 2022).

Overall, while different drug combinations with ivermectin have been evaluated, most offer limited therapeutic advantage over ivermectin monotherapy. This emphasizes the need for novel and more effect and truly synergistic treatment strategies for onchocerciasis.

##### 6.2.2.5 Other combination therapies

While ivermectin-based regimens dominate filariasis control, several other investigational drug combinations have been explored. However, most of them have been evaluated for lymphatic filariasis but not for onchocerciasis. Some of these include; moxidectin + albendazole, moxidectin + DEC + albendazole (Bjerum et al., 2023). Unfortunately, these combinations showed no benefit and are likely unsuitable for evaluation in onchocerciasis. In contrast, other combinations with albendazole have yielded encouraging results; notably, a 7-day rifampicin plus albendazole regimen achieved more than 99% *Wolbachia* depletion and accelerated macrofilaricidal activity (Turner et al., 2017). Similarly, a combination of doxycycline and albendazole (given for 3weeks and 3days respectively) increased the proportion of female worms with degenerated embryogenesis (Klarmann-Schulz et al., 2017). More recently, AWZ1066S combined with benzimidazoles (albendazole or oxfendazole) demonstrated synergistic anti-*Wolbachia* effects across multiple rodent filariasis models, achieving rapid (>90%) *Wolbachia* depletion in 5 days (Hegde et al., 2024). A short course regimen of AWZ1066S-albendazole combination produced prolonged embryogenesis inhibition and partial adulticidal effects, resulting in complete transmission blockade (Hegde et al., 2024). Together, these findings suggest that specific albendazole-containing combinations may reduce treatment duration and improve efficacy. However, further evaluations are pending.

Current research is shifting towards prophylactic treatment approaches. For instance, a combination of emodepside and oxfendazole may improve their therapeutic potential targeting multiple stages of the parasite (Jawahar et al., 2021). This underscores the need for a deeper understanding of emodepside’s mode of action, which could inform the development of more effective and targeted strategies for the treatment and elimination of onchocerciasis.

#### 6.2.3 Drugs in development

Despite efforts towards the development of effective anthelmintics for onchocerciasis, there are still significant concerns. The absence of drugs that have macrofilaricidal effects and the emergence of resistance to ivermectin, the current preferred treatment option, underscores the urgency for safer and more effective onchocercal therapeutics. In parallel, research is shifting towards prophylactic treatment approaches. For instance, a combination of emodepside and oxfendazole may improve their therapeutic potential targeting multiple stages of the parasite (Jawahar et al., 2021). These advances highlight the translational potential of veterinary-derived therapies, and justify further evaluation of emodepside and oxfendazole as candidates for repurposing in onchocerciasis.

##### 6.2.3.1 Oxfendazole

Oxfendazole is a benzimidazole methylcarbamate, and like the benzimidazoles, they selectively bind to parasite β-tubulin inhibiting critical processes necessary for the parasite’s survival (Lubega and Prichard, 1990, 1991; Enejoh and Suleiman, 2017). Used against gastrointestinal cestodes and nematodes in various animals, oxfendazole is approved in the US for the treatment and control of lung worms and intestinal worms in cattle (FDA, 2017; Gonzalez et al., 2019). Oxfendazole is the only anthelmintic registered for the treatment of porcine cysticercosis and the first licensed intervention against epilepsy in the developing world (WHO, 2017). In *in vitro* and *in vivo* studies, oxfendazole showed both macrofilaricidal and microfilaricidal activity against filarial worms (Hübner et al., 2020; Gokool et al., 2024). Additionally, Pionnier et al. (2019) reported that oxfendazole lacked activity against *Loa loa* microfilariae in mice, a desirable feature for use in regions where *Loa loa* co-infection complicates onchocerciasis treatment (Pionnier et al., 2019; Risch et al., 2024). Together, these results have encouraged its repurposing for onchocerciasis. A Phase I multiple ascending dose study in healthy adults was completed and showed a favorable safety profile (Bach et al., 2020). Oxfendazole has now progressed to Phase II proof-of-concept study to examine its safety and efficacy in treating multiple helminthic infections including onchocerciasis (Dällenbach, 2017; Esteves, 2025). If successful, oxfendazole could represent a pivotal advancement in the fight against onchocerciasis, offering a safe alternative particularly in *Loa loa* co-endemic regions.

##### 6.2.3.2 Emodepside

Emodepside is a semi-synthetic derivative of PF1022A, a cyclooctadepsipeptide originally isolated in the 1990s from the fungus *Mycelia sterilia* (*Rosellinia* sp.*)*, found on *Camellia japonica* leaves (Sasaki et al., 1992; Jeschke et al., 2005; Epe and Kaminsky, 2013). Initially produced by researchers from Japan, and later by Bayer Animal Health, it was developed for use in veterinary medicine and marketed for cats and dogs in Europe (Sasaki et al., 1992; Gillon et al., 2021). Emodepside’s macrofilaricidal potency and broad-spectrum of anthelmintic activity attracted interest in further development for onchocercal therapy. Collaborative drug discovery efforts targeting neglected tropical diseases, particularly onchocerciasis later identified emodepside as a potential candidate for human use (Krücken et al., 2021). Its broad spectrum of activity against the different stages of filarial nematodes, confers a notable advantage over other anthelmintics (Hübner et al., 2021).

The low toxicity of emodepside in animals and its success in clinical trials for hookworm and whipworm encouraged its investigation for onchocerciasis (Mrimi et al., 2023; Njeshi et al., 2024). Phase I clinical trials for onchocerciasis showed that it was safe and well tolerated with no major adverse reactions (Gillon et al., 2021). Phase II trials began in 2021, and results are awaited, with a primary end date of September 2026 (DNNi, 2024). Emodepside is orally bioavailable, suitable for tablet formulation, has a long half-life, and shows minimal side effects (Gillon et al., 2021). These properties make it promising for use in mass drug administration programs, offering hope for safety in *Loa loa* co-endemic regions.

Emodepside acts through a unique mechanism, targeting the latrophilin-like receptor (LAT-1), a G-protein-coupled receptor, and SLO-1K, a calcium-activated potassium channel (Saeger et al., 2001; Buxton et al., 2011; Kashyap et al., 2019; Krücken et al., 2021; Njeshi et al., 2024). While its effect on the latrophilin-like receptors inhibits pharyngeal pumping, its effect on SLO-1 channels causes hyperpolarization leading to paralysis and eventual death of the parasite. Further studies suggested that the SLO-1K channels are the major target for emodepside activity. Emodepside inhibited pharyngeal pumping but not locomotion in double mutants of lat-1 and lat-2 in *Caenorhabditis elegans*, confirming that emodepside acts directly on SLO-1K channels (Guest et al., 2007). Kulke et al., 2014 further showed in *Xenopus* oocyte experiments that emodepside can directly activate SLO-1K channels (Kulke et al., 2014). Nevertheless, the contribution of latrophilin receptors in emodepside’s mode of action cannot be completely ruled out. The human orthologue for SLO-1, KCNMA, shows reduced sensitivity to emodepside making it selective for the parasite (Crisford et al., 2011, 2015). Therefore, emodepside could emerge as a significant advancement towards achieving onchocerciasis elimination efforts.

### 6.3 Resistance

Pharmacological resistance is the inherent decline in a population’s responsiveness to a drug that was previously efficacious at a conventional dose. It arises when the organism or its life stages evolve mechanisms to survive, develop and reproduce despite repeated exposure to therapeutic concentrations that would typically be lethal or inhibitory. Drug resistance develops primarily through processes of evolution and natural selection, which can also be driven by human-mediated factors such as prolonged monotherapy, subtherapeutic dosing and poor treatment compliance.

In the context of onchocerciasis, ivermectin has long served as a cornerstone of MDA programs. However, over the past two decades, there have been increasing concerns about the emergence of resistance as suboptimal responses have been reported in endemic regions. Researchers have identified several indicators suggestive of emerging resistance including: (1) faster-than-expected repopulation of microfilaria in the skin post-treatment; (2) reduced microfilaria suppression after multiple rounds of treatment; (3) observable phenotypic resistance and (4) genetic alterations within the parasite population.

(Borsboom et al., 2003) reported a significant resurgence of onchocerciasis within a few years of treatment interruption, despite repeated ivermectin administration in some foci in Cameroon. Similarly, Osei-Atweneboana et al. documented an accelerated repopulation of microfilariae in the skin after treatment with ivermectin in endemic communities in Ghana. More recently, Abong et al. (2020) observed reduced clearance and increased repopulation rate post ivermectin treatment in Bafia, an endemic region in Cameroon (Abong et al., 2020), further raising concerns over ivermectin’s declining efficacy.

The presence of adult female worms resistant to the anti-fecundity effects of multiple rounds of ivermectin treatment also point to the emergence of phenotypic resistance (Osei-Atweneboana et al., 2007, 2011). Bourguinat et al., 2007 linked these observations to genetic selection in the female parasites. Their study involved the genetic analysis of the β-tubulin gene and revealed a significant selection for β-tubulin heterozygotes over homozygotes in female worms following a three-year ivermectin treatment period. Interestingly, they found that, the homozygous females were markedly more fertile than the heterozygotes. Their findings align with earlier reports that linked ivermectin resistance to the selection of specific β-tubulin genes (Eng and Prichard, 2005; Eng et al., 2006), and are further supported by subsequent research showing a direct interaction between ivermectin and tubulins (Ashraf et al., 2015). Thus, the occurrence of the selected β-tubulin genes may confer ivermectin tolerance and parasite survival under pressure. Moreover, ivermectin resistance *O. volvulus* has also been linked to the selection of specific P-glycoprotein genes (Eng and Prichard, 2005; Blancfuney et al., 2025). Studies in other parasites have corroborated these findings (Xu et al., 1998; Le Jambre et al., 1999; Peachey et al., 2017). By actively pumping out ivermectin, P-glycoprotein can significantly contribute to reducing its efficacy and hence contribute to resistance. However, contrary to these findings, Doyle et al. (2017) proposed that the suboptimal response was due to a genetic drift and not a direct selection pressure (Doyle et al., 2017). Using pooled next generation sequencing, they characterized genetic diversity within adult female worm populations with different ivermectin treatment history and responses, from Cameroon and Ghana. Multiple regions of the genome associated with the treatment responses (good response and suboptimal response) were identified. Their analysis suggested that ivermectin responsiveness is a polygenic quantitative trait, influenced by shared or related molecular pathways, rather than individual gene mutations alone.

In conclusion, the growing evidence of ivermectin resistance across endemic regions emphasizes the urgent need for enhanced surveillance, genetic monitoring and the development of diversified treatment strategies.

## 7 Vaccine development

Although no vaccine for human onchocerciasis is currently available, several promising candidates are advancing. Recent approaches have focused on subunit vaccine candidates that target *O. volvulus* larval stages, with *Ov*-103 and *Ov*-RAL-2 emerging as promising lead antigen targets (Abraham et al., 2021). A formulation of both antigens combined with the Advax-2 adjuvant was shown to induce Th1, Th2 and Th17 cytokine responses and significantly reduce infective larval survival in mouse cell lines (Ryan et al., 2021). The Onchocerciasis Vaccine for Africa Initiative (TOVA) endorsed the bivalent vaccine (composed of Ov-103 and Ov-RAL-2) for clinical trial in naturally infected cattle in Cameroon, where it exhibited favorable safety profiles and the ability to induce strong protective responses (Zhan et al., 2022; Nebangwa et al., 2024). Additionally, a fusion protein *Ov*-FUS-1, which combines *Ov*-103 and *Ov*-RAL-2 with adjuvants Advax-CpG, alum, or AlT4, elicited high IgG titers in mice and non-human primates and conferred protective immunity by killing the larvae (Ryan et al., 2023). Other lead vaccine candidates include Ov-CPI-2 (Ov7), OV9M, Ov-ALT-1, Ov-B20, Ov-TMY-1, Ov-CHI-1, OvB8, Ov-FAR-1, and Ov-FBA, Ov-ASP-1 (Zhan et al., 2022). Furthermore, reverse vaccinology, which screens pathogen genomes to identify potential vaccine-target proteins, has also identified 23 promising candidates recommended for experimental validation (Sheyin et al., 2021).

In addition, multi-epitope subunit vaccine candidates with potential prophylactic and therapeutic effects and possible cross-protection in related nematodes are in development (Shey et al., 2021). One example Ov-DKR-2, which combines both B- and T-lymphocyte epitopes of 8 immunogenic vaccine antigens showed strong reactivity with antibodies from sera of *O. volvulus* infected individuals, endemic normals and people with loiasis (Shey et al., 2021). In a subsequent study, Shey et al. (2022) designed another multi-epitope vaccine that integrated B- and T-cell epitopes from 14 different proteins, with in silico analysis showing a robust immune response (Shey et al., 2022). Kwarteng et al. (2021) also developed a construct that fused CD8^+^, CD4^+^, and B-cell epitopes with the RS-09 adjuvant, which simulated strong antibody and memory T-cell responses in silico (Kwarteng et al., 2021).

Together, these findings support the continued development of engineered epitope-based vaccines as promising tools for onchocerciasis control.

## 8 Future directions in onchocerciasis research

Recent years have seen important advances in onchocerciasis research, including improved diagnostic techniques, development of anti-Wolbachia strategies, combination therapy, novel macrofilaricidal candidates, and emerging vaccine platforms. Despite this progress, none of these approaches has yet resulted in sustained control at the population level, especially in endemic setting, highlighting critical knowledge gaps. Key challenges include;

- the need for enhanced diagnostics
- development of safe and more effective macrofilaricidal therapies suitable for mass drug administration,
- improved understanding of ivermectin resistance mechanisms
- the absence of a licensed vaccines.

In addition, the pathogenesis of onchocerciasis-associated neurological disorders remains unresolved.

Future research should therefore prioritize the development of compounds with macrofilaricidal activity suitable for mass drug administration programs, alongside improved diagnostics to better detect worm burden and monitor treatment response. Furthermore, integration of immunological, genomic, and epidemiological approaches will be essential to clarify neurological disease mechanisms. Finally, continued investment in vaccine development and implementation research will be indispensable for achieving long-term transmission interruption and eventual elimination of onchocerciasis.

## 9 Conclusion

Onchocerciasis continues to pose significant therapeutic and health challenges, despite control efforts. Its pathogenesis is intricately tied to the host’s immune response to dying microfilariae and the presence of *Wolbachia*, its bacterial symbiont. Together, these factors account for most of the disease’s clinical burden. Although diagnosis and treatment have advanced over the years, the limitations encountered with ivermectin its inability to kill adult worms remains an unresolved limitation.

Current advances in improved therapies and vaccine development including subunit and in silico-designed multi-epitope vaccines show promising progress. However, these strategies are still in preliminary stages, and none are yet widely available. Moving forward, a more comprehensive approach that integrates improved diagnostics, enhanced treatment options, and preventive tools like vaccines will be critical. Addressing these gaps is essential to achieving sustainable elimination of onchocerciasis and improving outcomes for affected populations.
